# Gastric Carcinogenesis and Underlying Molecular Mechanisms: *Helicobacter pylori* and Novel Targeted Therapy

**DOI:** 10.1155/2015/794378

**Published:** 2015-04-07

**Authors:** Toshihiro Nishizawa, Hidekazu Suzuki

**Affiliations:** ^1^Division of Gastroenterology and Hepatology, Department of Internal Medicine, Keio University School of Medicine, 35 Shinanomachi, Shinjuku-ku, Tokyo 160-8582, Japan; ^2^Division of Research and Development for Minimally Invasive Treatment, Cancer Center, Keio University School of Medicine, 35 Shinanomachi, Shinjuku-ku, Tokyo 160-8582, Japan

## Abstract

The oxygen-derived free radicals that are released from activated neutrophils are one of the cytotoxic factors of *Helicobacter pylori*-induced gastric mucosal injury. Increased cytidine deaminase activity in *H. pylori*-infected gastric tissues promotes the accumulation of various mutations and might promote gastric carcinogenesis. Cytotoxin-associated gene A (CagA) is delivered into gastric epithelial cells via bacterial type IV secretion system, and it causes inflammation and activation of oncogenic pathways. *H. pylori* infection induces epigenetic transformations, such as aberrant promoter methylation in tumor-suppressor genes. Aberrant expression of microRNAs is also reportedly linked to gastric tumorogenesis. Moreover, recent advances in molecular targeting therapies provided a new interesting weapon to treat advanced gastric cancer through anti-human epidermal growth factor receptor 2 (HER-2) therapies. This updated review article highlights possible mechanisms of gastric carcinogenesis including *H. pylori*-associated factors.

## 1. Introduction

*Helicobacter pylori* infection is one of the most prevalent infectious diseases worldwide and 40–50% of the global human population is estimated to be infected.* H. pylori *has been identified as a group 1 carcinogen by the World Health Organization International Agency for Research on Cancer (WHO/IARC, 69372 Lyon CEDEX 08, France) and is associated with the development of noncardia gastric cancer. Eradication of* H. pylori *infection has been reported as an effective strategy for both the treatment of peptic ulcers and gastric mucosa-associated lymphoid tissue (MALT) lymphoma as well as prevention of gastric cancer [1–3].

A randomized controlled trial (RCT) in Japan demonstrated significant prophylactic effects of* H. pylori *eradication on the development of metachronous gastric carcinoma after endoscopic resection [1]. According to a systemic review of 15 papers,* H. pylori *eradication significantly reduces the prevalence of gastric cancer by approximately one-third [4]. A recent meta-analysis of RCTs also shows that* H. pylori *eradication seems to reduce gastric cancer risk [relative risk (RR): 0.65, 95% confidence interval (CI): 0.43–0.98] [5]. In 2009, the committee of the Japanese Society for Helicobacter Research (JSHR) revised the guidelines for diagnosis and treatment of* H. pylori* infection [6].* H. pylori *eradication therapy achieved a strong recommendation, because it is useful for the treatment of gastric or duodenal ulcers, treatment and prevention of* H. pylori*-associated diseases such as gastric cancer, and inhibition of the spread of* H. pylori* infection.

Bacterial virulence factors, such as, cytotoxin-associated gene A (CagA), cause inflammation and activate oncogenic pathways. Activated neutrophils are the main source of reactive oxygen species (ROS) and reactive nitrogen species production in* H. pylori*-infected stomachs. Excessive oxidative stress can damage DNA in gastric epithelial cells, indicating its possible involvement in gastric carcinogenesis [7]. Gastric cancer arises from multiple genetic and epigenetic alterations in oncogenes, tumor-suppressor genes, cell cycle regulators, cell-adhesion molecules, and DNA repair genes.

Recent studies provided evidence that expression of an aberrant DNA/RNA editing enzyme, activation-induced cytidine deaminase (AID), might be a mechanism of mutation accumulation in the gastric mucosa during* H. pylori*-associated gastric carcinogenesis. The roles of microRNAs are increasingly apparent, and aberrant expression of microRNAs may contribute to the development and progression of gastric cancer. Moreover, recent advances in molecular therapies provided a new interesting weapon to treat advanced gastric cancer through anti-human epidermal growth factor receptor 2 (HER-2) therapies.

Consequently, this paper summarizes the molecular mechanism of* H. pylori*-associated gastric carcinogenesis.

## 2. Oxidative Stress

Oxygen-derived free radicals that are released from activated neutrophils are considered potential toxic factors that contribute to* H. pylori*-induced gastric mucosal injury. Neutrophils express myeloperoxidase, which produces the hypochlorous anion (OCl^−^). OCl^−^ reacts with the ammonia (NH_3_), which is produced from urea by* H. pylori*-associated urease, to yield a lipophilic, highly cytotoxic oxidant, monochloramine (NH_2_Cl), that freely penetrates biological membranes to oxidize intracellular components [8]. Free radicals, including ROS and reactive nitrogen species, can bind with nucleic acids, turning them into mutated forms that play a role in multistep carcinogenesis [9].

The CD44 variant (CD44v), which is a cell-surface marker, that is associated with cancer stem-like cells, interacts with a glutamate-cystine transporter and controls the intracellular level of reduced glutathione (GSH) [10]. Human gastrointestinal cancer cells with a high level of CD44 expression revealed an enhanced capacity for glutathione synthesis and defense against ROS. These findings indicate that cancer stem-like cells with CD44v expression could have an ROS defense system that results from glutathione synthesis.

*H. pylori *upregulates spermine oxidase (SMOX) in gastric epithelial cells. SMOX metabolizes the polyamine spermine into spermidine and generates H_2_O_2_, which causes apoptosis and DNA damage. However, a subpopulation of SMOX^high^ cells is resistant to apoptosis, despite their high levels of DNA damage. Chaturvedi et al. reported that activation of epidermal growth factor receptor (EGFR) and erythroblastic leukemia-associated viral oncogene B (ErbB2) by* H. pylori* results in survival of gastric epithelial cells with DNA damage [11].

## 3. CagA

Epidemiological evidence indicates that* H. pylori* strains containing CagA are more virulent. CagA-positive* H. pylori* increases the risk of both intestinal and diffuse types of noncardia gastric cancer [12]. The CagA protein of* H. pylori*, which is delivered into gastric epithelial cells via bacterial type IV secretion system, is an oncoprotein that can induce malignant neoplasms. After the CagA protein is injected into the host cell cytoplasm, the EPIYA (Glu-Pro-Ile-Tyr-Ala) motif of CagA is tyrosine-phosphorylated by host Src kinases and subsequently changes the gastric epithelial morphology [13]. CagA binding to the protooncogene Src homology 2-containing protein tyrosine phosphatase (SHP-2) causes aberrant activation of SHP-2 and consequently of the ERK-MAPK (mitogen-activated protein kinase) pathway, which has been reported to play a role in carcinogenesis by inducing mitogenic responses [14]. East Asian CagA that contains the EPIYA-D motif demonstrates higher affinity for SHP-2 than Western CagA. These findings are consistent with the fact that East Asian strains dominate in countries with the highest rates of gastric cancer [15].

**Figure 1:**
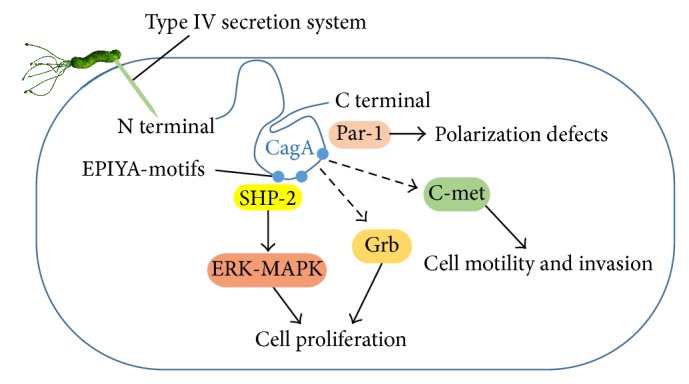
CagA and major functions. CagA is delivered into gastric epithelial cells via bacterial type IV secretion system. CagA interacts with many signaling molecules that are important for the regulation of cell proliferation, polarity, and motility.

On the other hand, CagA interacts with many signaling molecules (e.g., Per-1, c-Abl, Crk proteins, Grb proteins, and c-Met) that are important for the regulation of cell proliferation, scattering, and morphology (Figure 1). CagA, that is translocated into the host cell, is degraded by autophagy and short-lived. However, CagA, that is translocated into CD44v9-positive gastric cancer stem-like cells, which are characterized by ROS resistance that results from their rich GSH content, is thought to escape ROS-dependent autophagy, resulting in gastric carcinogenesis [16].

## 4. AID

**Figure 2:**
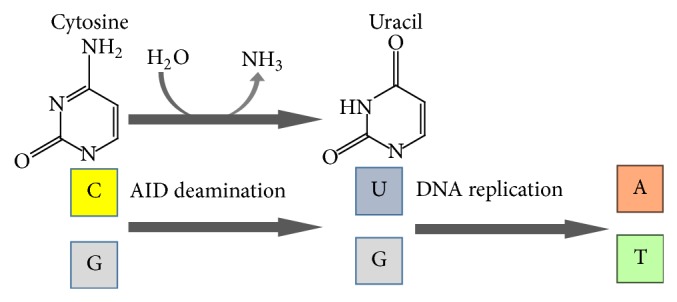
Activation-induced cytidine deaminase (AID) as the DNA/RNA editing enzyme. AID is induced by* H. pylori* infection and promotes the accumulation of various mutations.

The DNA/RNA editing enzyme, AID, is a 198 amino acid protein that directly converts cytosine into uracil (Figure 2). AID is specifically induced in germinal center B cells to carry out somatic hypermutation and class-switch recombination, which are two processes that are responsible for antibody diversification. Because of its mutagenic potential, AID expression and activity are tightly regulated to minimize unwanted DNA damage. Surprisingly, AID is also induced by inflammation and microbial infections in nonimmune cells.

*H. pylori *infection mediates aberrant AID expression in gastric mucosal epithelial cells. Additionally, AID expression was shown to be triggered by proinflammatory cytokines, such as TNF-*α* or IL-1, and correlated with mononuclear cell infiltration and intestinal metaplasia. After eradication of* H. pylori*, the level of AID expression decreases [17]. Strong evidence indicates that CagA-positive* H. pylori*-mediated upregulation of AID resulted in the accumulation of nucleotide alterations in the TP53 tumor suppressor gene in gastric cells [18]. Whole-exome sequencing revealed that somatic mutations accumulated in various genes in inflamed gastric tissues. The mutations that accumulated in gastric tumors as well as gastric mucosal tissues with* H. pylori*-induced gastritis were predominantly C : G > T :  A transitions in GpCpX motifs, which are markers of cytidine deamination that are induced by AID [19]. Increased cytidine deaminase activity in* H. pylori*-infected gastric tissues appears to promote the accumulation of various mutations and might promote gastric carcinogenesis.

## 5. Oncogenes

Mutational activation and/or amplification of several oncogenes such as ErbB, KRAS, PIK3CA, MET, and MYC has been documented in gastric cancer.

Epidermal growth factor receptor (EGFR), a member of ErbB receptor family, is involved in the regulation of gastric mucosal cell proliferation and progression of gastric cancer. Overexpression of ErbB1 (EGFR) and ErbB2 (HER-2) is found in 27–64% and 6–34% of gastric cancer, respectively [20]. Activation triggers a cascade of events that involves autophosphorylation and activation of tyrosine kinase domain, Ras/Raf/MAPK pathway, phospholipase C-*γ*, and phosphatidylinositol-3-kinase (PI3K)/Akt/mammalian target of rapamycin (mTOR). Ras, an oncogenic small GTPase, has three isoforms, namely, K-Ras, H-ras, and N-ras. Constitutive activation of Ras and Ras-related proteins promotes cell proliferation and increase invasion and metastasis while inhibiting apoptotic cell death.* K-RAS* gene was found to be mutated (codon-12) in intestinal-type cancer, but not in diffuse-type cancer [21].* K-RAS* gene mutations in* H. pylori*-associated chronic gastritis are more frequent in gastric cancer patients than in cancer-free patients, suggesting that K-RAS mutation may be involved in the early stage of gastric carcinogenesis [22].

Trastuzumab is a monoclonal antibody that binds to extracellular domain of the receptor, acting by blockage of the HER-2 receptor cleavage, inhibition of dimerization, and the induction of antibody-dependent cellular cytotoxicity (ADCC). In phase III ToGA trial, the addition of trastuzumab to standard cisplatin and 5-fluorouracil improved overall survival from 11.1 to 13.8 months in patients with HER-2 amplified gastric adenocarcinomas [23]. In phase III EXPAND trial, the addition of cetuximab (chimeric monoclonal anti-EGFR antibody) to standard cisplatin and capecitabine did not improve progression free survival in patients with advanced gastric cancer [24]. Similarly, in phase III REAL-3 trial, the addition of panitumumab (fully humanized monoclonal anti-EGFR antibody) to epirubicin, oxaliplatin, and capecitabine did not improve overall survival in patients with advanced gastric adenocarcinoma [25]. In addition, retrospective biomarker analysis has failed to identify a clear patient subset that would derive benefit from EGFR-directed therapies. Lapatinib is dual tyrosine kinase inhibitor active on EGFR and HER-2. In phase III TYTAN trial, the addition of lapatinib to weekly paclitaxel in second-line setting improved overall response rate in patients with HER-2-positive gastric adenocarcinomas (odds ratio, 3.85; 95% CI, 1.80–8.87, *P* < 0.001) but did not significantly improve overall survival (lapatinib+paclitaxel, 11.0 versus paclitaxel, 8.9 months; *P* = 0.104) [26].

Phase III GRANITE-1 study evaluated the efficacy of mTOR inhibitor everolimus compared to the best supportive care in patients with advanced gastric cancer that progressed after initial chemotherapy. In this study, median survival was reportedly 5.39 months with everolimus versus 4.34 months with placebo (HR = 0.90; 95% CI, 0.75–1.08) [27]. Patients in this study were not preselected with respect to PI3K/AKT/mTOR pathway alteration.

## 6. Tumor-Suppressor Genes

The* p53* tumor-suppressor gene, the guardian of human genome, is frequently inactivated in the tissue of gastric cancer as well as in preneoplastic lesions, by loss of heterozygosity (LOH), missense mutation, or frame-shift deletions [12]. The p53, 53 kDa phosphoprotein, is a transcription factor for including DNA repair genes in response to DNA damage. Activation of p53 also arrests the cell cycle to allow enough time for fixation of DNA damage. However, if DNA damage is beyond repair, p53 induces apoptotic cell death. The mutations of* p53* have also been identified in gastric adenoma and intestinal metaplasia. We have recently reported that p53 downregulation due to increased MDM2-phosphorylation induces autophagy, which causes CagA oncoprotein degradation translocated from the bacterial body of* H. pylori* to gastric epithelial cells and then inhibits mTOR [16].

APC is a multidomain protein with binding sites for numerous proteins including Wnt signaling pathway. APC plays major role in cell adhesion, cell migration, spindle formation, and chromosome segregation [28].* APC* mutations are the second most frequent mutations in gastric cancer and have been observed in 30–40% of intestinal type cancer and in less than 2% of diffuse type cancer [29]. The mutations of* APC* have also been identified in gastric adenoma and intestinal metaplasia, indicating that they occur during preneoplastic stage of gastric cancer development. LOH and mutations of phosphatase and tensin homolog (*PTEN*) were observed in gastric cancers as well as in precancerous lesions [30].

## 7. DNA Methylation

**Figure 3:**
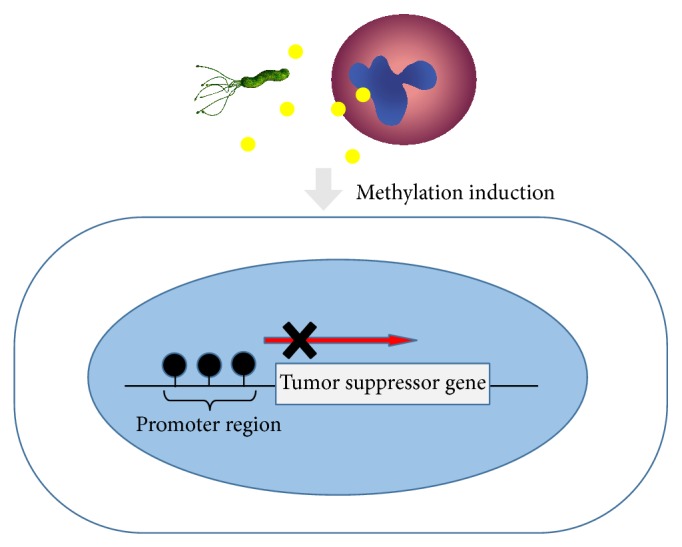
DNA methylation inactivates tumor suppressor genes without gene mutation. Chronic inflammation by* H. pylori* infection induces aberrant promoter methylation in tumor-suppressor genes.

Methylation of CpG islands in a promoter region inhibits gene transcription by interfering with transcriptional initiation and serves as an alternative mechanism of inactivating tumor suppressor genes without gene mutation. Among factors known to cause aberrant DNA methylation, aging and chronic inflammation are known to promote the accumulation of DNA methylation.* H. pylori *infection induces aberrant promoter methylation in tumor-suppressor genes, including* p16*,* LOX*, and* CDH1*. Furthermore, a number of tumor suppressor genes, including* hMLM1*,* p14*,* p15*,* GSTP1*,* RASSF1*,* COX-2*,* APC*,* CDH4*,* DAP-K*,* THBS1*,* TIMP-3*,* RARβ*,* MGMT*,* CHFR*,* DCC*,* RUNX3*, and* TSLC1*, are known to be silenced by hypermethylation in gastric cancer [31] (Figure 3).

Recent meta-analysis revealed that the frequencies of* p16* promoter methylation in gastric cancer tissue were higher than those of normal and adjacent tissues [Normal: odds ratio (OR) = 23.04, 95% CI = 13.55–39.15, *P* < 0.001; Adjacent: OR = 4.42, 95% CI = 1.66–11.76, *P* = 0.003]. Furthermore, significant associations of* p16* promoter methylation with TNM stage, histologic grade, invasive grade, and lymph node metastasis are shown (TNM stage: OR = 3.60, 95% CI: 2.17–5.98, *P* < 0.001; Histologic grade: OR = 2.63, 95% CI: 1.55–4.45, *P* < 0.001; Invasive grade: OR = 3.44, 95% CI: 1.68–7.06, *P* = 0.001; Lymph node metastasis: OR = 2.68, 95% CI: 1.66–4.32, *P* < 0.001) [32].

Forkhead box (Fox) proteins comprise an evolutionarily conserved family of transcriptional regulators. In particular, FOXD3 bound directly to the promoters and activated transcription of genes that encode the cell death regulators CYFIP2 and RARB. Recent methylation profile analyses revealed that FOXD3-mediated transcriptional control of tumor suppressors is deregulated by* H. pylori* infection-induced hypermethylation [33]. Alternatively, CagA enhanced DNA methyltransferase 3B and enhancer of zeste homologue 2 expression, which resulted in the attenuation of* let-7* expression by histone and DNA methylation [34]. Aberrant epigenetic silencing of* let-7* expression leads to Ras upregulation.

DNA methylation levels of specific CpG islands are associated with risk of gastric cancer. Nanjo et al. identified seven novel gastric cancer risk markers that reflect epigenomic damage, that is induced by* H. pylori* infection, and the hypermethylated CpG islands had high ORs (12.7–36.0) in an analysis [35]. Moreover, 5-aza-2′-deoxycytidine (5-aza-dC) is a DNA demethylating agent. Niwa et al. showed that 5-aza-dC treatment can prevent* H. pylori*-induced gastric cancers in the Mongolian gerbil model [36]. Removal of induced DNA methylation and/or suppression of DNA methylation induction could become a target for prevention of chronic inflammation-associated cancers.

## 8. Angiogenesis

Elevated concentrations of vascular endothelial growth factor (VEGF) have been described in patients with advanced gastric cancer and correlated with decreased survival. VEGF and VEGF receptors are overexpressed in 36–40% of gastric cancer. Phase III AVAGAST and AVATAR studies have evaluated the addition of bevacizumab (anti-VEGF-A monoclonal antibody) to first-line platinum-fluoropyrimidine chemotherapy in advanced gastric cancer patients but failed to demonstrate improved survival [37, 38].

Phase III REGARD trial evaluated the efficacy of ramucirumab, a monoclonal antibody VEGFR-2 antagonist, compared to the best supportive care (BSC) in patients with advanced gastric cancer that progressed after previous chemotherapy. Median survival was 5.2 months with ramucirumab versus 3.8 months with placebo (HR = 0.776; 95% CI 0.603–0.998; *P* = 0.047) [39]. Based on data from this REGARD trial, the Food and Drug Administration (FDA) in United States approved ramucirumab as second-line therapy for patients with advanced gastric cancer.

Apatinib, inhibitor of VEGF-2, showed improved progression free survival and overall survival in heavily pretreated patients with metastatic gastric cancer in phase II trial [40]. Phase III trial of apatinib is ongoing.

## 9. MicroRNAs

MicroRNAs are noncoding RNAs that are comprised of 18–24 nucleotides that can posttranscriptionally downregulate various target genes. Recent studies have shown that a considerable number of microRNAs are altered following infection with* H. pylori*, and specific microRNA deregulation was found to contribute to host inflammation, cell-cycle progression, inhibition of apoptosis, cell invasion, and metastasis [41]. MicroRNAs that function as oncogenes, including* miR-17*,* miR-21*, and* miR-106a*, were upregulated, whereas microRNAs that function as tumor suppressors, such as* miR-101*,* miR-181*,* miR-449*,* miR-486*, and* let-7a*, were downregulated in gastric cancer [42, 43].

Shiotani et al. reported that the expression of oncogenic microRNAs (*miR-17/92* and the* miR-106b-93-25* cluster,* miR-21*,* miR-194*, and* miR-196*) is significantly higher in the intestinal than nonintestinal metaplasia.* H. pylori *eradication improves microRNA deregulation, but not in the intestinal metaplasia. MicroRNA deregulation is not completely reversible by eradication alone in long-term* H. pylori* colonization [44].

Runt domain transcription factor 3 (RUNX3) is a tumor suppressor, that is silenced in cancer via hypermethylation of its promoter. Previously, we reported that* H. pylori* eradication significantly increases RUNX3 expression in gastric epithelial cells [45]. Lai et al. reported that* miR-130b* expression is upregulated in gastric cancer, and this is inversely associated with* Runx3* hypermethylation [46].* miR-130b* overexpression increases cell viability and reduces cell death following the downregulation of RUNX3 protein expression. Recently, Wang et al. reported that* miR-301a* is upregulated in gastric cancer and directly downregulates RUNX3 expression [47].

Furthermore,* microR-34b/c* is considered a tumor suppressor and transcriptional target of p53. Based on a scheduled follow-up study of endoscopic biopsy from noncancerous mucosa in the gastric body from 129 patients after curative endoscopic resection of gastric cancer, the cumulative incidence of metachronous gastric cancer was significantly higher among patients with elevated miR-34b/c methylation, indicating that methylation of* miR-34b/c* in the gastric mucosa may be a useful biomarker for predicting the risk of metachronous gastric cancer [48].

## 10. Cancer Stem Cell

Cancer stem cells have been defined as a unique subpopulation in tumors that possess the ability to initiate tumor growth and sustain tumor self-renewal. Gastric cancer stem cells were first isolated and identified in 2009. Takaishi et al. identified CD44 as a gastric cancer stem cell marker as CD44+ cells were able to form into spheroid colonies in serum-free media* in vitro* and gastric tumors after xenografts in nude mice* in vivo*, whereas CD44-negative sorted cells were not [49]. These cells have the ability for self-regeneration and resistance for chemotherapy- or radiation-induced cell death. Cancer stem cells-targeted therapy is a novel direction for treating and preventing gastric cancer. In our recent study, translocated CagA from* H. pylori* to the epithelial cells could accumulate in CD44v9-expressing gastric cancer stem-like cells by escaping the autophagic degradation pathway due to their characteristics of xCT-dependent ROS resistance [16].

The high expression of CD44 is positively correlated with malignant transformation, metastasis, and relapse of gastric cancer [50]. We previously reported that the recurrence rate of early gastric cancer was significantly higher in the CD44v9-positive than the CD44v9-negative cohorts (hazard ratio, 21.8; 95% CI, 5.71–83.1) [42]. CD44v9 expression in the tissue of primary gastric cancer represents a potential predictive marker for recurrence.

Additionally, there is another hypothesis. It is thought that gastric cancer stem cells are derived from bone-marrow-derived mesenchymal stem cells. When there is injury, the bone-marrow-derived mesenchymal stem cells can mobilize from the bone marrow and participate in tissue repair. Varon et al. labeled bone-marrow-derived cells with green fluorescent protein in mice model of infection with* H. pylori*. After 1 year, gastric glands that contained green fluorescent protein-positive cells were detected in 90% of mice infected with* H. pylori*. Almost 25% of high-grade dysplastic lesions included cells originating from the bone marrow [51]. The results suggest that bone-marrow-derived mesenchymal stem cells are the source of gastric cancer.

## 11. Conclusion

As shown in this review, molecular mechanisms of gastric carcinogenesis have been extensively studied. Alterations in multiple genes and complex copy number and gene expression profiles have been identified in gastric cancer over the two decades. New strategies had been developed for advanced gastric cancer treatment. However, the response rates remain in the 25–40% range across published trials, and novel molecularly directed approaches are needed.
